# An intronic micro-deletion impacts the transcription and translation of *PKD1* gene

**DOI:** 10.3389/fgene.2025.1707053

**Published:** 2026-01-23

**Authors:** Wei Zheng, Xinli Xing, Xuejing Sun, Na Wei

**Affiliations:** 1 Department of Perinatology, Maternal and Child Healthcare of Dongchangfu County, Liaocheng, China; 2 Laboratory Department, Dongchangfu District Maternal and Child Health Hospital, Liaocheng, China

**Keywords:** genetic counseling, intronic variant, minigene splicing assay, *PKD1*, polycystic kidney disease

## Abstract

Polycystin-1 (PC1), encoded by the *PKD1* gene, forms a complex with polycystin-2 (*PKD2*; 173910) that regulates multiple signaling pathways to maintain normal renal tubular structure and function. Mutations in the *PKD1* gene are the primary cause of type 1 PKD (polycystic kidney disease), accounting for 78%–85% of all PKD cases. In this study, we report a case of a boy presenting with microscopic hematuria with multiple renal cysts and carrying an unreported intronic variant, c.12445-34_12445-10del, in the *PKD1* gene inherited from his father who also presented PKD. Sanger sequencing and reverse transcription polymerase chain reaction (RT-PCR) for minigene splicing assays showed two abnormal splicing alterations with the c.12445-34_12445-10del variant at the mRNA level: one causes a 16-bp deletion in exon 46, resulting in premature protein termination (p.Phe4149GlyfsTer45), and the other results in a 205-bp deletion, leading to delayed termination (p.Phe4149ProfsTer139). Based on the clinical characteristics and gene mutations with functional verification, the patient was finally diagnosed with PKD caused by PKD1 function defection, as confirmed by the combined clinical features and genetic analysis. Management strategies include dietary management, blood pressure monitoring, and regular follow-up of kidney function. This is the first study to report an intronic deletion in the *PKD1* gene that influences alternative splicing. Our findings expand the mutation spectrum leading to PKD1-related diseases and highlight the importance of genetic counseling for the family.

## 1 Introduction

Autosomal dominant polycystic kidney disease (ADPKD) is the most common inherited kidney disorder, with an estimated prevalence of 1 in 400 to 1,000 individuals (Chebib et al., 2025; Menezes and Germino, 2019). More than 15 genes have now been clearly identified in association with polycystic kidney disease (PKD), with autosomal dominant inheritance, involving six genes (*PKD1*, *PKD2*, *GANAB*, *DNAJB11*, *HNF1B*, and *NEK8*). Of the six genes, mutations in the *PKD1* gene (located at 16p13.3) are the primary cause of type 1 PKD, accounting for 78%–85% of all PKD cases. Type 1 PKD caused by the *PKD1* gene typically manifests more severely than that caused by *PKD2* mutations resulting in type 2 PKD; approximately 50% of PKD1 patients progress to end-stage renal disease (ESRD) by age 60, contributing to approximately 10% of all ESRD cases (Padovano et al., 2018; Casarella et al., 2022). The *PKD1* gene encodes polycystin-1 (PC1), a large transmembrane protein comprising 4,303 amino acids, whose structure includes an N-terminal extracellular region (containing multiple immunoglobulin-like PKD domains that mediate the cell–matrix interactions), a transmembrane domain (with 11 transmembrane-spanning segments forming an ion channel-regulating complex), and a C-terminal intracellular tail (involved in regulating signaling pathways such as Wnt and STAT); PC1 forms the polycystin complex (PC1/PC2) with polycystin-2 (PC2), encoded by the *PKD2* gene, localized to the ciliary–centrosomal complex where it functions as a mechanosensor regulating calcium signaling and cellular homeostasis (Lea et al., 2023; Hackmann et al., 2005; Su et al., 2018). The type of *PKD1* mutation significantly impacts disease progression: truncating mutations lead to ESRD at a mean age of 53 years, approximately 15 years earlier than non-truncating mutations; biallelic mutations are associated with increased familial mortality before age 65; and *de novo* mutations constitute approximately 6% of cases (Wei et al., 2024; Cornec-Le Gall et al., 2013).

There is currently no radical cure for PKD caused by simple gene mutations, and the treatment focuses on relieving symptoms and delaying disease progression. Targeted therapy has achieved breakthroughs, with several drugs targeting pathways related to disease-causing genes entering clinical application, which can inhibit cyst growth and protect renal function. In addition, supportive care is indispensable, including a low-salt diet, avoidance of nephrotoxic substances, and regular monitoring of renal function; patients in the end stage need to rely on dialysis or kidney transplantation to sustain life (Li et al., 2022; van Aerts et al., 2019; Torres et al., 2025; Masyuk et al., 2022; Wang et al., 2020; Bajwa et al., 2001; Kanaan et al., 2014).

In this study, we report a family in which both the father and son presented with PKD and carried an unreported intronic variant, c.12445-34_12445-10del, in the *PKD1* gene. We have verified that intronic deletion could result in two abnormal splicing alterations.

## 2 Materials and methods

### 2.1 Compliance with ethical standards

The research protocol received approval from the Medical Ethics Committee of Maternal and Child Healthcare of Dongchangfu County (ethical approval no. 20250802), ensuring compliance with ethical standards. Prior to any clinical/laboratory procedures, the research team acquired written informed consent from the participants or their parents/guardians for pediatric subjects. All study procedures fully adhered to the ethical principles outlined in the Declaration of Helsinki.

### 2.2 Next-generation sequencing (NGS)

All three family members underwent whole-exome sequencing (WES). Genomic DNA was extracted from blood samples using the QIAamp DNA blood midi kit (Qiagen, Shanghai, China) and quantified using a NanoDrop 2000 spectrophotometer (Thermo Fisher Scientific, USA). Next-generation sequencing (NGS) was performed on the Illumina NovaSeq 6000 platform (USA). Target enrichment utilized the GenCap MedE006 capture kit (MyGenostics, Beijing, China) to screen for variants in the probands. WES achieved a mean coverage exceeding 95% across target regions (>10× coverage) with an average depth surpassing 100×. Sequencing reads were aligned to the GRCh37/hg19 human genome reference. Copy-number variation (CNV) analysis was also performed. Variants with minor allele frequencies exceeding 5% were filtered using the 1000 Genomes (https://www.internationalgenome.org/; phase 3), ESP6500 (http://evs.gs.washington.edu/EVS/; 20141222), ExAC (http://exac.broadinstitute.org; 20151129), gnomAD (https://gnomad.broadinstitute.org/; v2.1.1), and in-house databases (300,000 WES, MyGenostics). Standardized Human Phenotype Ontology (HPO) terms were prioritized for the analysis of likely pathogenic variants. The pathogenicity of novel variants was assessed using the software tools variant MutationTaster (https://www.mutationtaster.org/), Revel (https://sites.google.com/site/revelgenomics/), Human Splicing Finder Pro (https://www.genomnis.com/hsf), and SpliceAI (https://spliceailookup.broadinstitute.org/). Additionally, the reported variations were evaluated against the Human Gene Mutation Database (HGMD) (http://www.hgmd.org) and ClinVar (http://www.ncbi.nlm.nih.gov/clinvar/) databases. Variant pathogenicity was classified according to the American College of Medical Genetics and Genomics (ACMG) guidelines (Richards et al., 2015). Highly suspicious variants underwent confirmation via Sanger sequencing, followed by segregation analysis within families. Functional analysis of the variants incorporated information from the OMIM (http://www.omim.org) and dbSNP (https://www.ncbi.nlm.nih.gov/snp/) databases, the DECIPHER resource (https://www.deciphergenomics.org/), and relevant literature review.

### 2.3 mRNA extraction, reverse transcription, and PCR and sanger sequencing

Total RNA was extracted from cell pellets (5 × 10^6^ cells) using TRIzol reagent (Invitrogen, USA), with cells lysed in 500 µL TRIzol by vortexing. Following homogenization, chloroform was added to separate the mixture into aqueous and organic phases, with RNA retained in the aqueous phase. RNA was precipitated using isopropanol, washed, and its concentration quantified using a NanoDrop 2000 spectrophotometer (Thermo Fisher Scientific, USA). Complementary DNA (cDNA) was synthesized from the RNA template using a reverse transcription kit (R212, Vazyme). Specific cDNA fragments encompassing the variant site were amplified by PCR (primer sequences are provided in Supplementary Table 1). The resulting amplicons were analyzed via Sanger sequencing on an ABI Prism 3700 instrument (Applied Biosystems) to detect potential cDNA alterations arising from the variants.

### 2.4 Cell culture, plasmid transfection, and minigene assay

HEK293T cells were cultured in DMEM supplemented with 10% fetal bovine serum (FBS), with the medium replenished every 48 h. Upon reaching 90% confluence, cells were passaged at a 1:3 ratio. For transfection experiments, cells at 60% confluence were transfected with plasmid constructs using Lipofectamine 3000 reagent (Invitrogen, USA). The transfection mixture was replaced with fresh medium after 12 h to mitigate liposomal cytotoxicity. To evaluate variant-induced splicing alterations, minigene constructs were generated by amplifying specific PKD1 genomic regions: exon 45, part intron 45, and exon 46 from control gDNA. BamHI/XhoI-flanked primers were used for amplification (sequences provided in the Supplementary Material). Fragments were cloned into the pMini-CopGFP vector using the ClonExpress recombinant kit (Vazyme, Nanjing, China, #C112). Mutant plasmid was amplified using normal plasmids as templates according to the Mut Express Universal Fast Mutagenesis kit (Vazyme, Nanjing, China, #C216-01). Sequence-verified wild-type and mutant plasmids (Magen, Guangzhou, China) were transfected into HEK293T cells. The transfected cells were harvested 48 h post-transfection for total RNA extraction using TRIzol, followed by RT-PCR analysis with region-specific primers.

## 3 Results

### 3.1 Patients

The proband is a 2-year-and-2-month-old boy. Physical examination revealed microscopic hematuria, and renal ultrasound identified multiple renal cysts. He was otherwise asymptomatic. His mother is healthy; however, his father has a history of persistent microscopic hematuria and was found to have bilateral renal hypoplasia on renal ultrasound. The laboratory results for the proband and his parents are presented in Supplementary Table 2. His grandparents verbally stated that they were physically healthy and declined medical examinations.

### 3.2 Genetic analysis

**FIGURE 1:**
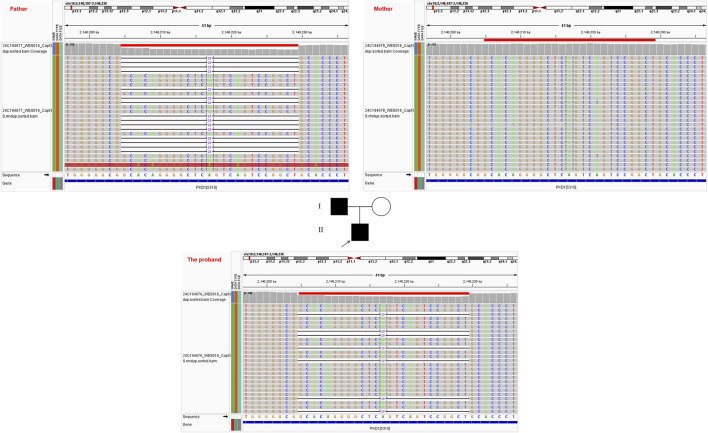
Pedigree chart of the family and the IGV (Integrative Genomics Viewer) screenshot containing the variant c.12445-34_12445-10del.

Clinical exome sequencing prioritized an intronic variant (c.12445-34_12445-10del) in the *PKD1* gene (NM_000215) as a candidate pathogenic variant in the proband presenting with ADPKD (OMIM 173900). The 25-bp deletion variant was absent from the population databases (1000 Genomes, gnomAD, ExAC, and ESP6500) and our in-house cohort. Segregation analysis via parental IGV (Integrative Genomics Viewer) screenshots from NGS data confirmed its paternal origin (Figure 1).

### 3.3 Bioinformatics analysis

The intronic variant was unreported, and its pathogenicity had not been studied further. Therefore, we predicted the pathogenicity of the variant using bioinformatics methods. First, neither intronic variant generates new exon cleavage sites, such as “GU–AG” or “AU–AC.” Moreover, the online RNA Splicer tool was used to assess the potential effects on the splicing of the variant. The delta scores for the intronic deletion, as predicted by the deep learning algorithm SpliceAI, were zero. Additionally, the Human Splicing Finder Pro tool showed that the intronic deletion variant could result in the creation of a new donor splice site.

### 3.4 Splicing study of the intronic variants using minigene assay

**FIGURE 2:**
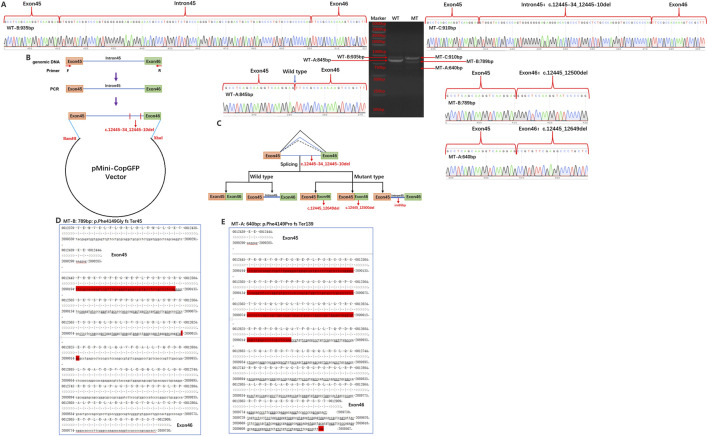
Splicing study of PKD1 c.12445-34_12445-10del variant by minigene assay. **(A)** Electrophoresis on agarose gels and Sanger sequencing of RT-PCR products based on the expression of the c.12445-34_12445-10del variant in wild-type and mutant-type plasmids. WT, wild type; MT, mutation type. **(B)** Schematic for the construction of the minigene plasmid. **(C)** Schematic of splicing for the PKD1 c.12445-34_12445-10del variant. **(D)** Schematic of splicing for the PKD1 c.12445-34_12445-10del variant from which the truncated protein p.Phe4149GlyfsTer45 (MT-B) arises. **(E)** Schematic of splicing for the PKD1 c.12445-34_12445-10del variant from which the truncated protein p.Phe4149ProfsTer139 (MT-A) arises.

We conducted the minigene analysis of the wild and mutant types carrying the *PKD1* intronic variant c.12445-34_12445-10del to characterize whether the abnormal splicing occurred. Agarose gel electrophoresis of RT-PCR products showed two bands from the wild type (935 bp, including intron 90 and 845 bp) and three bands from the mutant type (910 bp, including intron 65, 789, and 640 bp) (Figure 2A). Sanger sequencing was identified a normal splicing isoform (845 bp) and a transcript retaining an intronic sequence (935 bp) in the wild type. In contrast, the mutant exhibited two abnormal splicing alterations involving partial deletions of exon 46 (56 bp and 205 bp) and a transcript retaining an intronic sequence (910 bp) in the mutant type (Figure 2A–C). Our functional data therefore disagreed with the predictions generated by the online RNA Splicer tool and SpliceAI.

### 3.5 Analyzing the pathogenicity according to ACMG

In our study, we show that the variant c.12445-34_12445-10del could result in two types of abnormal truncated proteins (p.Phe4149GlyfsTer45 or p.Phe4149ProfsTer139) (PVS1) (Figure 2D–E). The frequency for the variant in the population database is zero (PM2), and the variant from the father with bilateral renal hypoplasia conforms to the genetic laws of dominant genetic diseases (PP4). According to the ACMG guidelines, the c.12445-34_12445-10del (p.Phe4149GlyfsTer45 or p.Phe4149ProfsTer139) variant (PVS1+PM2+PP4) was also classified as a pathogenic variant.

Combining the clinical findings and genetic analysis results, the patient was finally diagnosed with polycystic kidney disease caused by PKD1 function defection.

## 4 Discussion

ADPKD is predominantly a late-onset multisystem disorder characterized by bilateral renal cysts, hepatic cysts, and an elevated risk of intracranial aneurysms. Additional manifestations encompass cysts in the pancreas, seminal vesicles, and arachnoid membrane; aortic root dilatation and thoracic aortic dissection; mitral valve prolapse; and abdominal wall hernias. Renal involvement typically features early-onset hypertension, flank pain, and progressive renal insufficiency, with approximately half of affected individuals progressing to end-stage kidney disease (ESKD) by age 60 (Menezes and Germino, 2019; Boletta and Caplan, 2025; Perrone et al., 2015). The prevalence of hepatic cysts increases with age, occasionally leading to severe symptomatic polycystic liver disease (PLD), particularly in women. The prevalence of intracranial aneurysm is estimated to be five times higher than in the general population, and it is further elevated in those with a family history of aneurysms or hemorrhage (Gao et al., 2025). Significant inter-individual variability in the severity of renal disease and extra-renal manifestations. Diagnosis requires meeting age-specific renal imaging criteria in the proband combined with either an affected first-degree relative or molecular genetic identification of a heterozygous pathogenic variant in *PKD1* or *PKD2* or less commonly in *ALG5*, *ALG9*, *DNAJB11*, *GANAB*, or *IFT140*, among others (Chebib et al., 2025; Menezes and Germino, 2019; Lemoine et al., 2022; Chang et al., 2022; Devuyst, 2018; Zagorec et al., 2025). Genetically, PKD is usually autosomal dominant due to *PKD1* or *PKD2* variant; rarer genes or complex inheritance patterns (biallelic/digenic) occur in minorities (Chebib et al., 2025). Most patients inherit the variant, though 10%–20% arise *de novo*; offspring have a 50% inheritance risk. Prenatal/preimplantation genetic testing is feasible following pathogenic variant identification in the family (Chebib et al., 2025).

Mutations in the introns of *PKD1* are relatively rare, and their pathogenicity has been poorly validated (Cornec-Le Gall et al., 2013; Rossetti et al., 2012). This study found that the wild-type allele produces two transcript variants, whereas the mutant (MUT) allele generates three transcript variants. Among these, we speculate that the intron-containing variants were attributed to genomic contamination. The MUT allele leads to two truncated transcript variants: one causes a 16-bp deletion in exon 46, resulting in premature protein termination (p.Phe4149GlyfsTer45), and the other results in a 205-bp deletion, leading to delayed termination (p.Phe4149ProfsTer139).

**FIGURE 3:**
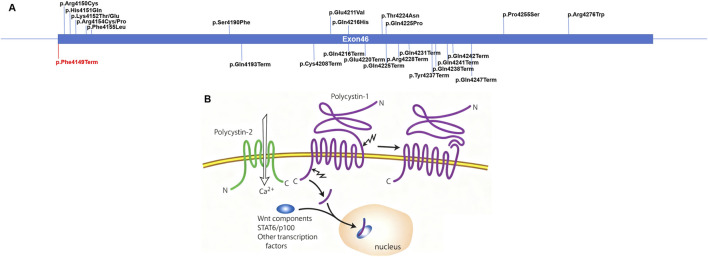
Schematic diagram for point mutations in exon 46 of JAK3 and the N- and C-terminal cleavages of the polycystin-1 (PC1) protein. **(A)** The reported 26 point mutations in JAK3 exon 46 are shown over the diagram. The variants reported are shown in blue, and the variant identified in this study is shown in red. The missense mutations are shown above, and the nonsense mutations are shown below. **(B)** The N-terminus of PC1 is cleaved at the G-protein-coupled receptor proteolytic site (GPS). Either of the two different cleavages can release C-terminal tail fragments that translocate to the nucleus with components of the Wnt pathway, STAT6/p100, and perhaps with other regulators of transcription (Gao et al., 2025).

Two abnormal proteins arise from frameshift mutations, leading to aberrant and premature termination along with truncation of the PKD1 C-terminus, which are specifically designated as p.Phe4149ProfsTer139 and p.Phe4149GlyfsTer45. Exon 46 serves as the final exon, encoding the amino acid sequence spanning 4,149 to 4,303 aa, which localizes to the CTT domain (4,078–4,303 aa). All exon 46 point mutations retrieved from the Human Gene Mutation Database (till 2024.04) have been mapped in Figure 3, which illustrates that mutations encompassing both truncating and point mutations that are positioned closer to the C-terminus are capable of inducing PKD. Furthermore, our literature review confirmed that the C-terminal domain of PKD1 is indispensable for its physiological functions (Higashihara et al., 2018; Chapin and Caplan, 2010); PKD1 encodes polycystin-1 (PC1), a 460-kDa protein featuring a large extracellular N-terminal region, a transmembrane segment, and a cytoplasmic C-terminal tail (CTT), whereas PKD2 encodes polycystin-2 (PC2), a member of the transient receptor potential family of non-selective cation channels. PC1 and PC2 interact via their CTT domains and co-localize to the primary cilium, where they may exert mechanosensory roles, with the ciliary trafficking of PC1 and formation of the PC1–PC2 complex being regulated by this interaction. PC1 also undergoes cleavage at its G-protein-coupled receptor proteolytic site (GPS), a process that is likely essential for its full functionality, and the CTT region of PC1 may undergo cleavage as well, with the resulting CTT translocating to the nucleus to initiate signaling (Figure 3B) (Chapin and Caplan, 2010; Onuchic et al., 2023). Notably, research has demonstrated that for carriers harboring PKD1 non-truncating mutations in the domain upstream of the GPS (GPS-upstream domain), transmembrane domain, or cytoplasmic CTT domain (4111–4303 aa), the mean renal survival times were 70.2, 67.0, and 50.1 years, respectively (p < 0.0001), indicating that renal survival shortens as mutation positions approach the CTT domain and underscoring the critical role of this domain in renal prognosis (Higashihara et al., 2018). Additionally, in the case of *PKD1* truncating mutations, substantial inactivation of nucleotides downstream of the mutation site is anticipated, implying that CTT domain inactivation occurs irrespective of the mutation’s specific location. The mean renal survival was significantly shorter in carriers of *PKD1* truncating mutations than in those with non-truncating mutations (p = 0.0348), with no significant difference in the mean renal survival observed between carriers of *PKD1* truncating mutations in the 3′-region and those in the 5′-region (p = 0.4375), whereas the mean renal survival was significantly shorter in carriers of *PKD1* non-truncating mutations in the 3′-region relative to those in the 5′-region (p = 0.0014) (Higashihara et al., 2018). These observations are hypothesized to stem from the variable extent of CTT domain inactivation, highlighting the potential pivotal role of the CTT domain in renal prognosis that warrants further validation in large-scale studies (Higashihara et al., 2018).

We also attempted to detect the gene splice variants of PKD1 in peripheral blood from both the father and son; however, PKD1 is not expressed in peripheral blood, leading to the failure of cDNA amplification. We also tried to detect PKD1 mRNA splice variants in urine, but both experiments were unsuccessful due to severe urine contamination and RNA degradation. If there is a discrepancy in the splice variants between the father and son, it may contribute to the observed renal phenotypic heterogeneity, which warrants further experimental verification. In addition, the accurate pathogenicity diagnosis for the family is pivotal to provide precise genetic counseling and perform prenatal diagnosis to deliver a healthy child.

## 5 Limitations and implications

The patient was found to carry the mutation since childhood; however, there are currently no effective drugs or treatments to prevent disease progression or to treat the father’s condition. This situation poses a significant psychological and financial burden on the family, highlighting an urgent need for the development of targeted therapies for PKD1-related disorders to address this clinical challenge.

## Data Availability

The original contributions presented in the study are publicly available. This data can be found in the National Omics Data Encyclopedia repository with the accession number OEZ00021767 at here: https://www.biosino.org/node/analysis/detail/OEZ00021767.
