# Supplementary material for: An intronic micro-deletion impacts the transcription and translation of PKD1 gene
Source: Front Genet. 2026 Jan 23;16:1707053. doi: 10.3389/fgene.2025.1707053 (PMC12875593; doi:10.3389/fgene.2025.1707053)
Supplement: Supplementary file 1 [file Table1.docx]

| **Supplementary Table 1. The primers sequences in the study** | |
| --- | --- |
| PKD1-RT-F： | GGCTAACTAGAGAACCCACTGCTTA |
| PKD1-RT-R： | CTAAGTGCTGCTGGGGTGGA |
| β-globin intron-F： | GATATACACTGTTTGAGATGAGGA |
| BGH-R： | TAGAAGGCACAGTCGAGG |

| **Supplementary Table 2. The laboratory results for the proband and his parents** | | | | |
| --- | --- | --- | --- | --- |
|  |  | **The proband** | **Father** | **mother** |
| **urine** | Leukocyte(LEU)(0-23) | 17.2 | 5.7 | 32 |
|  | Erythrocyte(RBC)(≤18) | 197 | 274 | 3 |
|  | occult blood(BLD)(NEG) | +++ | +++ | - |
|  | Proteinuria(PRO)(NEG) | - | - | - |
|  | ketone body(KET)(NEG) | - | - | - |
| **serum** | Creatinine(Cr) | 41 | 49 | 54 |
|  | Urea(2.7-7.1) | 4.17 | 4.08 | 3.21 |
|  | total protein(TP)(60-80) | 67.3 | 61.9 | 65.4 |
|  | Cholesterol(TC)(0-5.17) | 3.02 | 4.79 | 3.17 |
|  | HDL-C(0.8-1.5) | 1.47 | 0.93 | 1.09 |
|  | LDL-C(0-3.36) | 2.13 | 2.94 | 1.98 |
|  | Triglyceride(0-1.69) | 0.62 | 1.04 | 0.42 |
